# Strategy in talent systems: Top-down and bottom-up approaches

**DOI:** 10.3389/fspor.2022.988631

**Published:** 2022-08-16

**Authors:** Jamie Taylor, Áine MacNamara, Robin D. Taylor

**Affiliations:** ^1^School of Health and Human Performance, Faculty of Science and Health, Dublin City University, Dublin, Ireland; ^2^Grey Matters Performance Ltd., Stratford-upon-Avon, United Kingdom; ^3^Moray House School of Education and Sport, The University of Edinburgh, Edinburgh, United Kingdom

**Keywords:** talent development, Talent Development Environment, sport performance, elite performance, talent identification

## Abstract

Building on a large volume of recent research in talent identification and development, this paper future directions for research and practice. We suggest that strategic coherence become a greater point of emphasis in both, with the Performance, Outcome and Process framework holding the potential to signal various markers of effectiveness. Secondly, greater recognition of the need to deploy limited resources where they promote movement toward these markers of effectiveness. Finally, we make recommendations for the operationalising of strategy in talent and performance systems by considering the integration of top down and bottom-up strategic processes.

The last 20 years have seen a significant growth in talent development (TD) practice and research (Baker et al., 2020). Such interest has grown beyond the realm of practice and research, with significant media attention being placed on the pathways of elite athletes and the systems through which they develop. This attention has not always presented TD systems in a positive light (Calvin, 2017), with the suggestion that some talent systems have been built around the principles of industrialization, forcing conformity in athletes (Rothwell et al., 2018). Consequently, there is a growing emphasis on how talent systems can positively influence sporting culture beyond performance (Collins et al., 2012) and prevent harm coming to athletes (Bergeron et al., 2015). This has presented talent systems, and high-performance systems more broadly, with the challenge of understanding their ends beyond ultimate elite performance (cf. De Bosscher et al., 2015).

## Leading talent systems

The TD literature in sport has diverged into various communities of practice, built various conceptual models and adopted different foci, reflecting expertise studies more generally (Ward et al., 2019). One such focus has been investigating effective TD practice, as a means of informing the work of TD coaches and professionals. Initially in the British context (Martindale et al., 2005, 2007), then in Scandinavia (Henriksen et al., 2010a; Henriksen and Stambulova, 2017) the concept of the Talent Development Environment (TDE) was generated representing “all aspects of the coaching situation” that impact on the athlete's development (Martindale et al., 2005, p. 354). This body of research suggests that effective TDEs offer participants: long term aims and methods, wide ranging coherent messaging and support, appropriate and individualized development (Martindale et al., 2007), a range of factors that have now been tested across cultures (Ivarsson et al., 2015; Hall et al., 2021). In addition, features of ineffective environments have been examined, being characterized by a lack of integration, an incoherence of culture and short termism (Henriksen et al., 2014).

More recent work has begun to recognize that TD typically takes place across multiple settings, with barriers to effective practice often being systemic (Bjørndal and Ronglan, 2018; Taylor and Collins, 2021) necessitating greater attention on the coherence of athlete experience *through* their pathway (e.g., Curran et al., 2021). This coherence can be vertical, representing the level of difference between levels of performance (Webb et al., 2016) and horizontal, across a level, where multiple stakeholders can influence development (Taylor et al., 2021). To generate coherence for the athlete, it has been suggested that there is a need for integration within and outside of talent systems (Taylor and Collins, 2020). Integration being the extent to which various stakeholders work in tandem to support the athlete, vertically and horizontally (Taylor and Collins, 2022). From a strategic perspective, frameworks such as the Collaboration Success-Factors model have been suggested to enhance interorganisational practice (Mathorne et al., 2020). This approach aims to guide effective collaboration between organizations by identifying preconditions, the processes of, the reasons for, management of conflict and the expected benefits of collaboration (Mathorne et al., 2020). Given the potential for multiple organizations to influence practice, this is a welcome addition to the practitioner's toolkit. Yet, although there is recognition that incoherence of policy and practice is a key limiting factor (Henriksen et al., 2014; Taylor and Collins, 2021) there is little research that has examined, or can inform strategy in talent systems.

As such, here we make the case for a broader understanding of opportunity cost through the application of strategic lenses to talent systems. Therefore, we do not seek to present a new model of development (e.g., Bronfenbrenner and Morris, 2007; Bailey et al., 2010), instead, we refer to micro, meso and macro as lenses that help us understand levels of the broader system (e.g., Dopfer et al., 2004). Micro being the individual interactions that occur in day-to-day TD practice, meso representing collections of these micro systems, typically in the form of TDEs (Martindale et al., 2005), or individual organizations such as academies. Macro represents the interaction between organizations, typically at the national, or international level.

### Talent system strategy

Strategy is a concept with multiple definitions and perspectives on application (cf. Mintzberg et al., 2009). Here, we explicitly adopt the view that strategy can be seen as “the alignment of potentially unlimited aspirations with necessarily limited capabilities” (Gaddis, 2018, p. 21). One such area of strategic choice for talent systems is the timing and management of selection. To this point, rather than considering selection as a matter of resource allocation the literature has focused predominantly on systems making the “right” talent identification decisions, often with associated recommendations for making more accurate predictions. Yet the idea that athletes who are likely to “make it” can be identified from an early age has been roundly criticized (Abbott et al., 2005; Baker et al., 2018) and the current evidence base augments these arguments (Johnston and Baker, 2020) suggesting that selection is often more performance identification than talent identification (Baker et al., 2018). This is supported by the potential disadvantage conferred by early advantage, where those who are more likely to be selected early, are also more likely to be deselected later (McCarthy and Collins, 2014; McCarthy et al., 2022). At the macro level, this has led to critique of the “standard model of TD,” characterized by an emphasis on those identified by relative high performance and each vertical progression leading to large numbers of athletes being deselected (Bailey and Collins, 2013). Reflecting these selection processes, a recent case study of a recreational and competitive participation organization proposed the maxim “as many as possible, for as long as possible” as a means of countering these issues (Erikstad et al., 2021) and mitigating against the need to make accurate predictions (Collins and MacNamara, 2017).

This, however, leaves a number of issues. Whilst early high performance does not mean certain progression (e.g., Taylor and Collins, 2019), those who are better earlier are still more likely to progress than their peers (Bezuglov et al., 2022). In a world of limited resourcing, it is difficult to offer a high-quality development experience where individual needs are catered for (Martindale et al., 2007; Henriksen and Stambulova, 2017) and aspirations within and beyond the sporting context supported (Rongen et al., 2020; Williams and MacNamara, 2020). It is therefore beholden on talent systems to make decisions about who and when to select based on contextual demands and broader objectives, which will include factors beyond performance at the elite level (Collins et al., 2012; Bjørndal et al., 2017). Take for example the Norwegian national system, which following success at the 2022 Beijing Winter Olympics has been recognized as being highly effective (Bergeron et al., 2022). The system provides opportunities for large numbers of participants to engage at a high level of performance facilitated by a decentralized and loosely connected approach (e.g., Norwegian handball; Bjørndal and Ronglan, 2020). However, this system requires highly integrated practice (cf. Taylor and Collins, 2022) or risks incoherence for individual athletes (Bjørndal and Ronglan, 2018). Therefore, there is a need for both research and practice to recognize the obvious opportunity costs that face talent systems. This is especially important (and perhaps timely) with increasing pressure for legalistic defensibility of decision making (Johnston et al., 2021) and a range of philosophical positions beginning to question the dominant meritocratic cultural narratives (cf. Wooldridge, 2021).

#### Effectiveness

It is in this context that the constructs of efficiency and effectiveness have been applied to talent systems (Tucker, 2017). Given the long-term nature of TD, one of the key challenges in understanding effectiveness and the extent of overall “output” of the system is the difficulty of short-term measures. We therefore need to understand effectiveness on multiple levels, take for example: Performance, Outcome and Process (POP - Collins et al., 2019). Ultimate performance will be understood in terms of athlete progression, yet importantly, could also be judged by other criteria such as; contribution to the wider sport, or the extent to which athletes are prepared for life beyond HP. Outcomes can represent the key markers that athletes may need to achieve, or pass through, that signal longer term success. These could be the pragmatic milestones that athletes require for progression, often generated by funding agencies or the norms of the sport. For example, the number of athletes competing at world junior competitions, players achieving professional contracts, or in some contexts the value of transfer fees accrued. Finally, processes will include the features of effective TDEs, identified earlier (Martindale et al., 2010), along with markers such as the quality of coaching, the extent of integrated practice and coherence of athlete experience.

#### Efficiency

Of course, it is important to consider the broader culture which informs the allocation of resources and their social acceptability. Therefore, macro strategy needs to consider the norms of the sporting culture, sources and amount of funding, the number of participants in a sport, quality of coaching workforce and the different agendas that could impact TD. As such, efficiency represents the necessarily limited capabilities of all talent systems in terms of finance, time and attention. On the meso level, the need to show return on investment to investors, pressure for “quick” development for senior performance, and the extent to which the athlete is provided support to develop other areas of their life are some of the organizational agendas that are shaped by macro factors (Henriksen and Stambulova, 2017) and necessitate the need to work within various strategic parameters. Take for example the various strategic approaches that have characterized high effectiveness in performance national systems in Olympic/Paralympic sport (De Bosscher et al., 2016). Some have focused resource on a minority of athletes at the highest levels of performance, others spreading resource more broadly and integrating with participatory agendas.

At the micro level, individuals work within the context set by macro and meso factors, but still engage with a variety of strategic resource allocation decisions on a day-to-day basis. These can include the time limitations and attentional bandwidth for individual coaches and athletes. Importantly, a nested understanding and integration of different strategy levels is likely to be a critical feature of effective decision making at the micro level (Taylor and Collins, 2021) and prevent incoherence of organizational culture (Henriksen et al., 2014). For example, Güllich (2014) proposed the concept of the “individualistic approach” and the “collectivistic approach.” The former emphasising ongoing nurture and resource allocation to a limited number of athletes. The latter, where elite performers emerge from repeated selection and deselection. When a system is characterized by a collectivist approach, coaches need to understand their strategic function and coach in a manner that is supportive of a large breadth of athletes, rather than a limited number of favored individuals.

This has significant implications for both practice and research. Research has long acknowledged the range of complex biopsychosocial factors that influence TD (Abbott and Collins, 2004), mostly ending the reductionistic search for single variables causative of elite performance (Barraclough et al., 2022). We suggest that research at the system level should follow, recognizing the inherent complexity, perhaps utilizing mixed methods at multiple levels of analysis (Headley and Plano Clark, 2020). In turn, recommendations for “best practice” replaced by understanding of strategic context and evidence that informs practice (Neelen and Kirschner, 2020). In practice, no single model of athlete development can account for the way that TD *should* be done. This questions the application of age and stage specific frameworks being deployed outside the context of their development (MacNamara and Collins, 2014; Coutinho et al., 2016). Where talent systems choose to allocate their resources should be a matter of strategic intent, rather than a single way of doing things. To exemplify, whilst research may show a limited number of athletes progressing from junior international to the elite level, it doesn't necessarily follow that measures should be taken to enhance talent identification. In fact, doing so assumes that a low attrition from junior to senior is an important outcome marker, but may miss the macro picture, where repeated selection and deselection is desirable, especially if junior international experience isn't correlated with later elite performance (Herrebrøden and Bjørndal, 2022). In short, it is beholden on researchers to understand strategic context.

### Operationalising strategy

The previous section discussed the various strategic and resource allocation decisions necessary at all levels of talent systems (Maritan and Lee, 2017) and argued for greater recognition of trade-offs (cf. Kelly, 2009) and side effects (cf. Zhao, 2017). Given that strategy should consider the *relationship* between ends and means, we argue that systemic focus has fallen disproportionately on ends and means alone. The consequence being an overestimation of the impact of top-down strategic impetus (Moore, 2021). Whilst top-down agendas provide a bandwidth for activity, more attention should be paid to the interaction between bottom up and top-down processes. This means seeing the “bottom-up” as more than delivery of strategy and instead emphasizing the flexible working practices of expert practitioners (Stenhouse, 1975; Collins et al., 2015). As suggested by Mintzberg et al. (2009, p. 476):

Strategy formation is judgmental designing, intuitive visioning, and emergent learning; it has to be about transformation as well as perpetuation; it depends on individual cognition and social interaction, cooperation as well as conflict; it requires analyzing before and programming after as well as negotiating during; and all of this must be in response to what can be a demanding environment.

Strategy is therefore enabled by the integration of top-down and bottom-up functions through feedback loops and communication channels which allow for flexibility and course correction. More recent work has extended the concept of Active Inference (Friston et al., 2016) with centralized processes fundamentally intertwined with bottom-up input, toward the minimization of prediction error to iteratively optimize overall strategy and beliefs (Khezri, 2022). Top-down predictions are challenged from the bottom-up: “strategy is redefined as prediction processing that is subject to (bottom-up) stimuli-based error-minimization” (Khezri, 2022, pp. 4, 5). This challenges the view that the micro level simply needs to align with the macro, or that strategy is a top-down endeavor. Instead, agendas should be generated from top down and bottom up, with integration critical for effective deployment of limited resources. It is here that shared understanding of purpose is likely to enhance overall integration and therefore enhance the effective use of resources toward strategic ends (Mathorne et al., 2020; Taylor and Collins, 2021).

As a means of operationalising this integration, it has been proposed that developing shared mental models at all levels may be a vehicle for enhanced practice (Taylor and Collins, 2022). Thus, holding a clear understanding of the macro system agenda, the specific conditions of operation in an organization at the meso level and how that impacts day to day practice at the micro level. This should be generated by open sharing of information and co-construction, constructive conflict (Salas et al., 2005; Van den Bossche et al., 2011) and a reflexive approach (Van der Haar et al., 2013). Therefore, the nested integration of strategy depends on bi-directional open communication and the ongoing search for potentially divergent views (Tjosvold et al., 2014). A potential barrier being the power relations between the macro level (those who make decisions regarding resourcing either through finance or regulation) and those at the micro level who interface with athletes. Similarly, there can also be fractured communication between the so-called “ivory tower” and individual practitioners, especially if systems are of a significant size (e.g., at the national level). For the approaches advocated in this paper to be adopted, systems and processes need to be designed to minimize that distance.

### Example: Strategic parental engagement

As an example of the processes we advocate for, we discuss a single element of TD and how it may be approached based on differential resource allocation. One process marker of effectiveness at all levels, is the extent to which parents are leveraged as key stakeholders (Knight, 2019) and recognized as part of a triadic relationship (i.e., athlete-parent-coach; Henriksen et al., 2010b). The extent to which this relationship can be fostered depends on the financial and human resource available at each level of the system. Whilst optimal support for the athlete will involve significant communication with parents, it is a common complaint that coaches do not have enough time to do this on an individual basis. To exemplify the challenge, Table 1. provides an outline of top-down and bottom-up integration for TD parenting support. From a macro system perspective, at the low end of resourcing this could be developing generic parent support resources that can subsequently be adapted toward the needs of specific contexts. At the higher resourcing end, it could lead to the provision of ongoing educational support for TDEs. Higher meso level resourcing could be academy/sport led individualized psychology input, planned formal and informal communication, and bespoke induction processes for new parents. Lower end parental support could involve generic communication through social media and workshops. At the micro level, coaches need to consider the extent of their time allocation to each triad and weigh that up against other areas that might require their attention, whilst leveraging other inputs. At all levels, top-down beliefs and strategy should be tested by emergent problems, for example countering overly perfectionist parenting (Curran and Hill, 2022). Notably, at both ends of the resourcing spectrum, there is a need to weigh up the desired effect and the resource allocation put toward a given initiative. Whilst it may be effective, and indeed evidence informed, to engage with parents at the higher end, this resource could always have been deployed elsewhere.

**Table 1:** Integration of top-down and bottom-up approaches to parental engagement.

		**Resource intensive**	**Resource light**	**Considerations**	
Macro level – national system		Specific programmes of support built to help TDEs effectively engage with parents.	Wider promotion of the role of the parent in sport at the national level. Development of platform to guide parental engagement in and out of talent systems.	Must recognize the dilemmas of practice at the micro level. To offer value, needs to be age, stage and context appropriate. Overly restrictive and resource intensive approaches have potential to overly constrain meso/micro practice.	
Meso level–academy		Individual and bespoke parental engagement featured as part of all communications. Psychologist resource allocated to parents based on need.	Develop parent knowledge of talent development through ongoing workshops and communication (e.g., newsletters etc).	For optimal effect, needs to be coherent with macro/micro. Needs to be evidence informed and context specific.	
Micro level–the triad	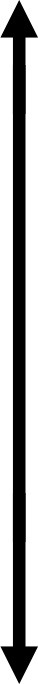	Planning for messaging and interaction with and through parents. Coaches engage in frequent, robust and open communication with parents.	Inviting parents to workshops and review meetings. Frequent generic communication (e.g., email, use of social media).	Cannot take up too much time to the detriment of other factors. Needs strong coach knowledge of individual triadic context and of effective TD parenting. Coaches feed in to meso level the typical challenges being faced.	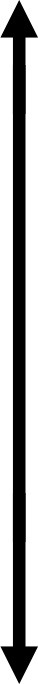

## Conclusion

This paper has presented an alternative approach to both talent system practice and research. We have proposed that strategic coherence is emphasised further, with the Performance, Outcome and Process framework used as a means to understand effectiveness. And, to direct resources in a manner coherent with overall strategy. In seeking to operationalise the concept of strategy in TD practice, we have suggested that top-down and bottom-up approaches to organizational development present opportunities for talent and performance systems. This presents a paradigm shift away from “upper echelon” strategy development, followed by “lower echelon” delivery (Khezri, 2022). Therefore, we have suggested the need for both top-down and bottom-up approaches, employing both deliberate and emergent processes (Mintzberg, 2007). As a necessity, these should be informed by open, honest dialogue and constructive conflict. These interactions may be facilitated by a greater understanding of the different markers of performance, outcome and process in talent systems (cf. Collins et al., 2019). We hope this perspective piece acts as a primer for future research and, importantly, for systems aiming for evidence informed practice.

## Data availability statement

The original contributions presented in the study are included in the article/supplementary material, further inquiries can be directed to the corresponding author.

## Author contributions

JT, ÁM, and RT contributed to conception of the paper and wrote sections of the manuscript. JT wrote the first draft of the manuscript. All authors contributed to manuscript revision, read, and approved the submitted version.

## Funding

Funding for open access publication through Dublin City University.

## Conflict of interest

Author JT was employed by Grey Matters Performance Ltd. The remaining authors declare that the research was conducted in the absence of any commercial or financial relationships that could be construed as a potential conflict of interest.

## Publisher's note

All claims expressed in this article are solely those of the authors and do not necessarily represent those of their affiliated organizations, or those of the publisher, the editors and the reviewers. Any product that may be evaluated in this article, or claim that may be made by its manufacturer, is not guaranteed or endorsed by the publisher.
